# Docosahexaenoic Acid Inhibits Tumor Promoter-Induced Urokinase-Type Plasminogen Activator Receptor by Suppressing PKCδ- and MAPKs-Mediated Pathways in ECV304 Human Endothelial Cells

**DOI:** 10.1371/journal.pone.0163395

**Published:** 2016-09-21

**Authors:** Sen Lian, Yong Xia, Thi Thinh Nguyen, Trong Thuan Ung, Hyun Joong Yoon, Nam Ho Kim, Kyung Keun Kim, Young Do Jung

**Affiliations:** Research Institute of Medical Sciences, Chonnam National University Medical School, Gwangju 501-190, Republic of Korea; University of South Alabama Mitchell Cancer Institute, UNITED STATES

## Abstract

The overexpression of urokinase-type plasminogen activator receptor (uPAR) is associated with inflammation and virtually all human cancers. Despite the fact that docosahexaenoic acid (DHA) has been reported to possess anti-inflammatory and anti-tumor properties, the negative regulation of uPAR by DHA is still undefined. Here, we investigated the effect of DHA on 12-*O*-tetradecanoylphorbol-13-acetate (TPA)-induced uPAR expression and the underlying molecular mechanisms in ECV304 human endothelial cells. DHA concentration-dependently inhibited TPA-induced uPAR. Specific inhibitors and mutagenesis studies showed that PKCδ, JNK1/2, Erk1/2, NF-κB, and AP-1 were critical for TPA-induced uPAR expression. Application of DHA suppressed TPA-induced translocation of PKCδ, activation of the JNK1/2 and Erk1/2 signaling pathways, and subsequent AP-1 and NF-κB transactivation. In conclusion, these observations suggest a novel role for DHA in reducing uPAR expression and cell invasion by inhibition of PKCδ, JNK1/2, and Erk1/2, and the reduction of AP-1 and NF-κB activation in ECV304 human endothelial cells.

## Introduction

Tumor metastasis is the most common cause of poor prognosis and deaths in cancer patients. Urokinase-type plasminogen activator (uPA) and urokinase-type plasminogen activator receptor (uPAR) system is thought to play a role in tumor angiogenesis [1] and tumor metastasis [2]. uPAR is over-expressed in tumors by multiple tumor-associated cell types including the tumor cells themselves, stromal cells and endothelial cells [3]. Coordination of extracellular matrix proteolysis and cell signaling by uPAR underlies its important function in tumor metastasis and make it an attractive therapeutic target in cancer [4]. uPAR appears to also elicit a plethora of cellular responses include cellular adhesion, differentiation, proliferation and migration [5, 6]. Moreover, uPAR expression increase with grade of tumor and maybe enriched in metastatic lesions [7]. The expression of a catalytically inactive enzyme or an antisense uPAR cDNA, which results in the decreasing of uPAR decreases cell invasiveness [8]. Therefore, agents with the ability to block uPAR expression may hold potential as treatments for human cancers. The level of uPAR expression is stimulated by a diverse set of agents, including vascular endothelial growth factor [9], epidermal growth factor [10], hepatocyte growth factor [11], and fibroblast growth factor [12] in a number of different cell types. uPAR is also stimulated by hypoxia in breast cancer cells [13].

TPA, a potent tumor promoter, stimulates renal tumor cell proliferation through activation of protein kinase C (PKC) [14]. TPA-induced uPAR is mediated by the activation of MAPK signaling pathways and transcription factors such as NF-κB and AP-1 in human ovarian and gastric cancer cells [15, 16]. Because of the critical roles of PKC, MAPKs, NF-κB, and AP-1 in TPA-induced uPAR expression and cancer cell metastasis, substances that inhibit these factors may confer anti-tumor activity.

Polyunsaturated fatty acids (PUFAs) can be divided into two major groups: ω-3 and ω-6 PUFAs [17]. Docosahexaenoic acid (DHA), a major ω-3 PUFAs that is enriched in fatty fish and fish oil supplements, is well known for its anti-inflammatory and anticancer properties [18, 19]. Regarding its anticancer effect, DHA was reported to inhibit MMP-9 expression in human breast cancer MCF-7 cells [20]. In addition, DHA has been shown to reduce monocyte chemoattractant-1 (MCP-1) through PPARγ and NF-κB in human epithelial cells [21].

In the present study, we aimed to investigate DHA’s effect on TPA-induced uPAR expression in ECV304 human endothelial cells, and to reveal its underlying molecular mechanisms.

## Materials and Methods

### Reagents

Dulbecco’s modified Eagle’s medium (DMEM), OPTI-modified Eagle’s medium, fetal bovine serum (FBS), phosphate buffered saline, and penicillin–streptomycin solution were obtained from HyClone (Logan, UT, USA). TrypLE^™^ Express was obtained from Gibco (Grand Island, NY, USA). The bicinchoninic acid protein assay kit was from Pierce (Rockford, IL, USA). TPA, DMSO, LY294002 hydrochloride, curcumin, rottlerin, and all other chemicals were purchased from Sigma-Aldrich (St. Louis, MO, USA). BAY11-7082, PD98059, SP600125, and SB203580 were purchased from Calbiochem (San Diego, CA, USA). Antibodies against uPAR, PKCδ, phos-PKCδ (Tyr 311), phos-Akt (Ser 473), Akt, phos-JNK1/2, JNK1/2, phos-Erk1/2, Erk1/2, phos-p38**,** p38, phos-c-jun, phos-c-fos, phos-p65 (Ser 536), phos-IκBα (Ser 32), and IкBα were purchased from Cell Signaling Technology (Danvers, MA, USA), and antibodies against c-jun, c-fos and Clathrin HC were purchased from Santa Cruz Biotechnology (Santa Cruz, CA, USA).

### Cell culture

The ECV304 human endothelial cell line was obtained from American Type Culture Collection (Manassas, VA, USA) and cultured in DMEM supplemented with 10% fetal bovine serum (FBS) and 0.6% penicillin–streptomycin at 37°C in a 5% CO_2_ humidified incubator. In these experiments, stimulants such as TPA, were added to serum-free media for the indicated time intervals. When the inhibitors were used, they were added 1 h before the TPA treatment.

### Cell viability assay

Cell viability after treatment was determined by the MTT assay. Cells were incubated with 1mg/ml MTT for 3 h, and subsequently solubilized in DMSO. The presence of DHA or the other chemicals did not interfere with the measurement at 570 nm wavelength measured using a microplate spectrophotometer (Epoch, Biotek, USA).

### Isolation of cell fractions

The cells were harvested and then washed twice with ice-cold PBS. Homogenization buffer A (200 μL; 20 mM Tris–HCl, pH8.0, 10 mM EGTA, 2 mM EDTA, 2 mM dithiothreitol, 1 mM phenylmethylsulfonyl fluoride, 25 μg/mL aprotinin, and 10 μg/mL leupeptin) was added to each dish, and the cells were scraped into a 1.5 mL tube. Cells were centrifuged at 5000× *g* for 15 min at 4°C. The cell pellet was collected as the nuclear fraction. The supernatant was centrifuged at 15,000×*g* at 4°C for 60 min to yield the pellet (membrane fraction) and the supernatant (cytosolic fraction).

### Reverse transcription PCR, and real-time PCR

Total cellular RNA was extracted from cells using RNAiso Reagent (TaKaRa Bio, Otsu, Japan). The complementary DNA was subjected to PCR amplification with the primer sets for glyceraldehyde 3-phosphate dehydrogenase (GAPDH) and uPAR, using a PCR master mix solution (iNtRON, Seongnam, Gyeonggi-do, Korea). The specific primer sequences were GAPDH sense, 5′-TTG TTG CCA TCA ATG ACCCC-3′; GAPDH antisense, 5′-TGA CAA AGT GGT CGT TGA GG-3′(836 bp); uPAR sense, 5′-CAC GAT CGT GCG CTT GTG GG-3′; and uPAR antisense, 5′-TGT TCT TCA GGG CTG CGG CA-3′ (285 bp). The PCR conditions included denaturation at 94°C for 30 s, annealing at 58°C for 30 s, and extension at 72°C for 45 s. The products were electrophoresed in a 1.5% agarose gel containing ethidium bromide. PCR product formation was monitored continuously during the reaction using Sequence Detection System software, version 1.7 (Applied Biosystems, Foster City, CA, USA). Accumulated PCR products were detected directly by monitoring the increase of the reporter dye (SYBR^®^). The mRNA expression levels of uPAR in the treated cells were compared to the expression levels in control cells at each time point using the comparative cycle threshold (Ct)-method [22]. The quantity of each transcript was calculated as described in the instrument manual and normalized to the amount of GAPDH, a housekeeping gene.

### Western blot analysis

After each experiment, cells were washed twice with cold PBS and were harvested in 100 μL of protein extraction solution (iNtRON, Seongnam, Gyeonggi-do, Korea). Cell homogenates were centrifuged at 10,000×*g* for 20 min at 4°C. Equal amounts of total cellular protein (50 μg) were electrophoresed in sodium dodecyl sulfate (SDS)-polyacrylamide gels, and the protein was then transferred to polyvinylidene difluoride membranes (Millipore, Billerica, MA, USA). Nonspecific binding sites on the membranes were blocked with 5% nonfat dry milk in 15 mM Tris/150 mM NaCl buffer (pH 7.4) at room temperature for 2 h. Membranes were incubated with target antibody. The membranes were then probed with secondary antibody labeled with horseradish peroxidase. The bands were visualized using an enhanced chemiluminescence kit (Millipore, Billerica, MA, USA) and were scanned by a luminescence image analyzer (Vilber Lourmat, France).

### Transient transfection with siRNAs and dominant negative mutants

Stealth RNAi duplexes corresponding to human siRNAs of PKC, PKCδ, and Akt were purchased from Santa Cruz Biotechnology (Santa Cruz, CA, USA). The plasmids encoding dominant negative mutants of MEK-1 (pMCL-K97M), JNK (pMCL-TAM67), and p38 MAPK (pMCL-mP38) were kindly provided by Dr. N.G. Ahn (University of Colorado, Boulder, CO, USA), Dr. M.J. Birrer (NCI, Rockville, MD, USA), and Dr. J. Han (Scripps Research Institute, CA, USA), respectively. The phosphorothioated double-stranded oligodeoxynucleotide (ODNs) with sequences targeting the AP-1 binding site (5'-CAC TCA GAA GTC ACT TC-3' and 3'-GAA GTG ACT TCT GAG CTG-5') were prepared (Genotech, St. Louis, MO, USA) and annealed (AP-1 decoy ODNs). The dominant negative mutants of I-κBα and I-κBβ and NIK were kindly provided by Dr. D.W. Ballard (Vanderbilt University, Nashville, TN, USA) and Dr. W.C. Greene (University of California, CA, USA), respectively. All mutants were prepared by using Qiagen (Valencia, CA, USA) plasmid DNA preparation kits. Transient transfections of siRNAs (100 nM) and dominant negative mutants (1 μg) were carried out using Lipofectamine 2000 from Invitrogen (Carlsbad, CA, USA).

### Measurement of uPAR, AP- 1 and NF-κB luciferase activity

The plasmid pGL3/uPAR-promoter was generous gift from Dr. Y. Wang (Australian National University, Canberra, Australia). The NF-κB and AP-1 luciferase reporter plasmid was purchased from Clontech (Palo Alto, CA, USA). ECV304 were seeded and grown until they reached 70% confluence. Then, cells were co-transfected with siRNAs of PKC, PKCδ, Akt, scrambled sequence, uPAR luciferase. PRL-TK was transfected as an internal control. Cells were collected with cell culture lysis reagent (Promega, Madison, WI, USA) and the luciferase activity was determined using a luminometer (Centro XS lb960 microplate luminometer,Berthold Technologies, USA) according to the manufacturer’s protocol.

### Matrigel invasion assay

The cell invasion assay was carried out using 10-well chemotaxis chambers (Neuro Probe, Gaithersburg, Maryland, USA) with an 8-μM pore membrane (Neuro Probe) in DMEM with 10% FBS as the chemoattractant in the lower chamber. The non-invading cells on the upper surface of each membrane were removed from the chamber by using cotton swabs, and the invading cells on the lower surface of each membrane were stained using the Quick-Diff stain kit (Becton-Dickinson, Franklin Lakes, NJ, USA). After two washes with water, the chambers were allowed to air dry. The number of invading cells was counted using a phase-contrast microscope.

### Statistics analysis

Data are shown as the mean ± standard deviation (SD) and represent the mean of at least three separate experiments performed in triplicate. Differences between data sets were determined by *t*-tests. Differences described as significant in the text correspond to *P* values of<0.05.

## Results

### DHA inhibits TPA-induced uPAR in ECV304 cells

**Fig 1:**
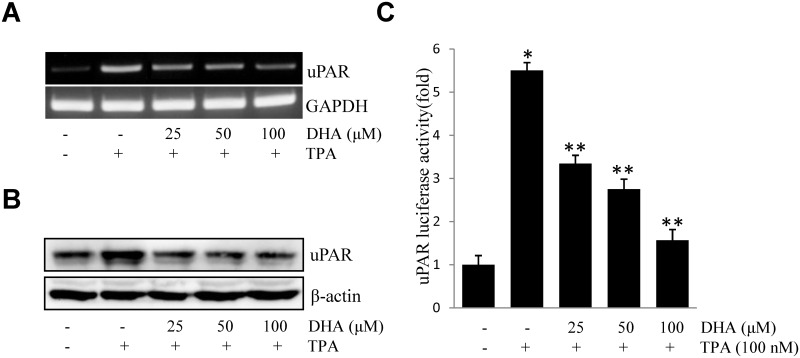
DHA inhibits TPA-induced uPAR in ECV304 cells. Cells were pretreated with DHA (25, 50, 100 μM) for 1 h, followed by incubation with 100 nM TPA for 4 or 16 h. uPAR mRNA level (A), protein level (B), and promoter activity (C) were measured by RT-PCR, western blot, and luciferase activity assay, respectively. *P<0.05 versus control; **P<0.05 versus TPA only. The data represent the mean ± SD from triplicate measurements.

To investigate the suppressive effect of DHA on the up-regulation of uPAR, ECV304 cells pretreated with DHA were incubated with TPA. TPA-stimulated uPAR mRNA expression (Fig 1A), protein expression (Fig 1B), and promoter activity (Fig 1C) were inhibited by DHA in a concentration-dependent manner as illustrated. These results suggested that DHA inhibited TPA-induced uPAR expression in ECV304 cells.

### DHA inhibits TPA-induced uPAR by suppressing PKCδ activation

**Fig 2:**
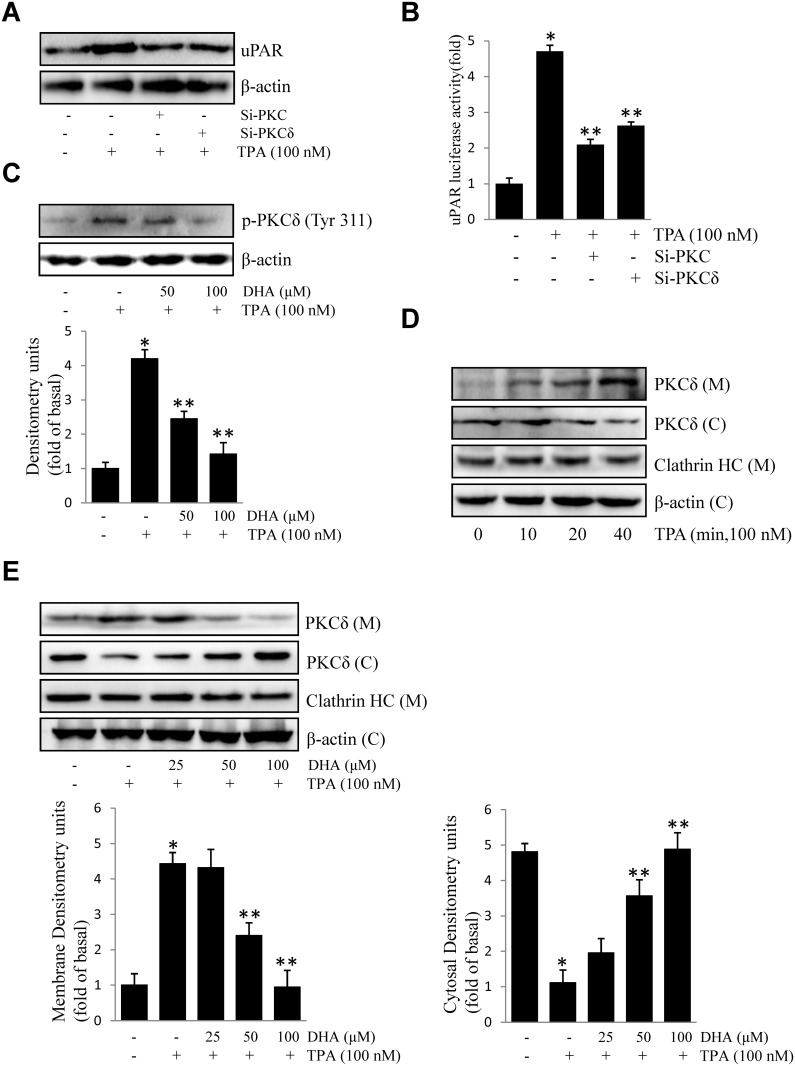
DHA inhibits TPA-induced uPAR via suppression of PKCδ. (A) si-PKC or si-PKCδ were transfected into cells. After incubation with 100 nM TPA for 16 h, uPAR levels were evaluated by western blotting. (B) si-PKC or si-PKCδ were co-transfected with PGL3-uPAR into cells. After incubation with 100 nM TPA for 4 h, luciferase activity was measured using a luminometer. (C) Cells were treated with DHA (50, 100 μM) followed by TPA for 20 min, and the phosphorylation of PKCδ was analyzed by western blotting. (D) Cells were treated with TPA for 10–40 min, the subcellular components of cells were extracted, and the levels of PKCδ in the cytosolic and membrane fractions were analyzed by western blotting. (E) Cells were treated with DHA (25, 50, 100 μM) followed by TPA for 20 min, and PKCδ in the cytosol and membrane was evaluated as described above. *P<0.05 versus control; **P<0.05 versus TPA only. The data represent the mean ± SD from triplicate measurements.

Activation of PKCs has been shown to correlate with tumor metastasis [23]. However, contributions of PKC isoforms to TPA-induced uPAR in ECV304 cells are still unclear. As shown in Fig 2A and 2B, transfection of si-PKC and si-PKCδ inhibited TPA-induced uPAR protein expression and promoter activity. Next, we found that DHA inhibits TPA-induced phosphorylation of PKCδ (Fig 2C). Activation of PKC by TPA involves in the translocation of PKC isoforms to the plasma membrane. Translocation of the PKCδ protein from the cytosol to the membrane was detected in TPA-treated cells, but was blocked by the addition of DHA (Fig 2D and 2E). This illustrated that PKCδ activation is involved in TPA-induced uPAR, which was inhibited by the addition of DHA.

### DHA suppresses Erk1/2 and JNK1/2 activation downstream of PKCδ

**Fig 3:**
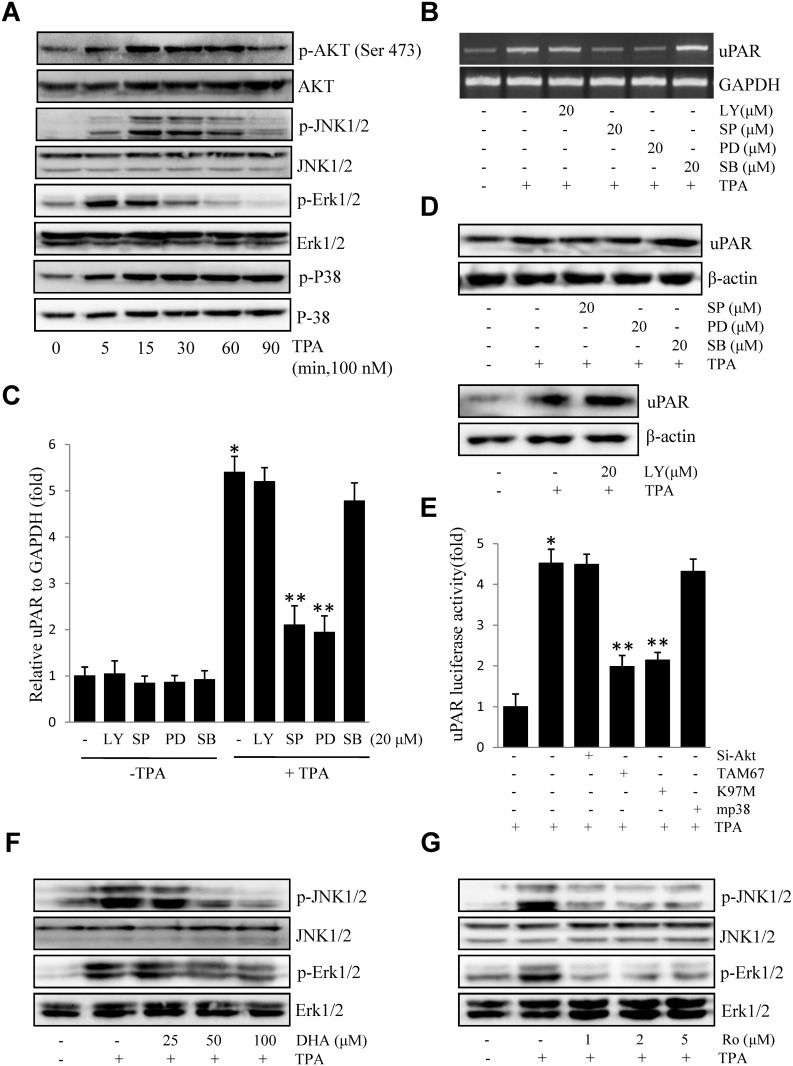
DHA inhibits TPA-induced uPAR via suppression of JNK1/2 and Erk1/2. (A) Cells were incubated with 100 nM TPA for 0–90 min, and cell lysates were blotted using specific antibodies. (B) Cells pretreated with LY (20 μM), SP (20 μM), PD (20 μM), or SB (20 μM) for 1h were incubated with 100 nM TPA for 4 h. After incubation, uPAR mRNA levels were determined by RT-PCR. (C) Cells were pretreated with LY (20 μM), SP (20 μM), PD (20 μM), or SB (20 μM) for 1 h in the presence or absence of 100 nM TPA for 4 h. After incubation, uPAR mRNA levels were determined by real-time PCR. (D) Cells pretreated with SP (20 μM), PD (20 μM), SB (20 μM) or LY (20 μM) for 1 h were incubated with 100 nM TPA for 16 h. After incubation, uPAR protein levels were determined by western blotting. (E) si-Akt, dominant negative mutants of JNK (TAM67), MEK-1(K97M), or mutant p38 MAPK (mp38) were co-transfected with PGL3-uPAR into cells. After incubation with 100 nM TPA for 4 h, luciferase activity was measured using a luminometer. (F) Cells were treated with DHA (25, 50 and 100 μM), followed by TPA treatment for 15 min, and cell lysates were blotted using specific antibodies. (G) Cells were treated with Ro (1, 2, and 5 μM), followed by TPA treatment for 15 min. Expressions of phosphorylated JNK1/2 and phosphorylated Erk1/2 were evaluated by western blotting. *P<0.05 versus control; **P<0.05 versus TPA only. The data represent the mean ± SD from triplicate measurements.

To determine the signaling molecules involved in TPA-induced uPAR expression, we investigated the levels of phosphorylated Akt and changes in MAPKs (Erk1/2, JNK1/2, and P38 MAPK) in ECV304 cells exposed to TPA for various periods. As shown in Fig 3A, induction of Akt, JNK1/2, and Erk1/2, and p38 phosphorylation elicited by TPA were detected. Pharmacological inhibitors of Akt and MAPK were used to determine the molecular mechanisms by which TPA induces uPAR expression. As shown in Fig 3B and 3C, treatment of SP (a JNK inhibitor) and PD (an Erk inhibitor) decreased TPA-induced uPAR mRNA expression, whereas treatment with LY or SB did not. As expected, treatment of SP and PD also decreased TPA-induced uPAR protein expression (Fig 3D). Consistent with these results, dominant negative mutants of JNK (TAM67) and MEK-1 (K97M) inhibited TPA-induced uPAR promoter activity. However, si-Akt or the dominant negative mutants of p38 MAPK (mp38) showed no effect (Fig 3E). These findings demonstrated that uPAR induction by TPA was mediated through JNK1/2 and Erk1/2 activation, and that these were blocked by pretreatment with DHA (Fig 3F). Furthermore, phosphorylation of JNK1/2 and Erk1/2 was blocked by treatment with rottlerin (a PKCδ inhibitor) (Fig 3G), suggesting that DHA suppression of JNK1/2 and Erk1/2 occurs downstream of PKCδ.

### DHA inhibits TPA-induced uPAR by suppressing DNA-binding activities of AP-1

**Fig 4:**
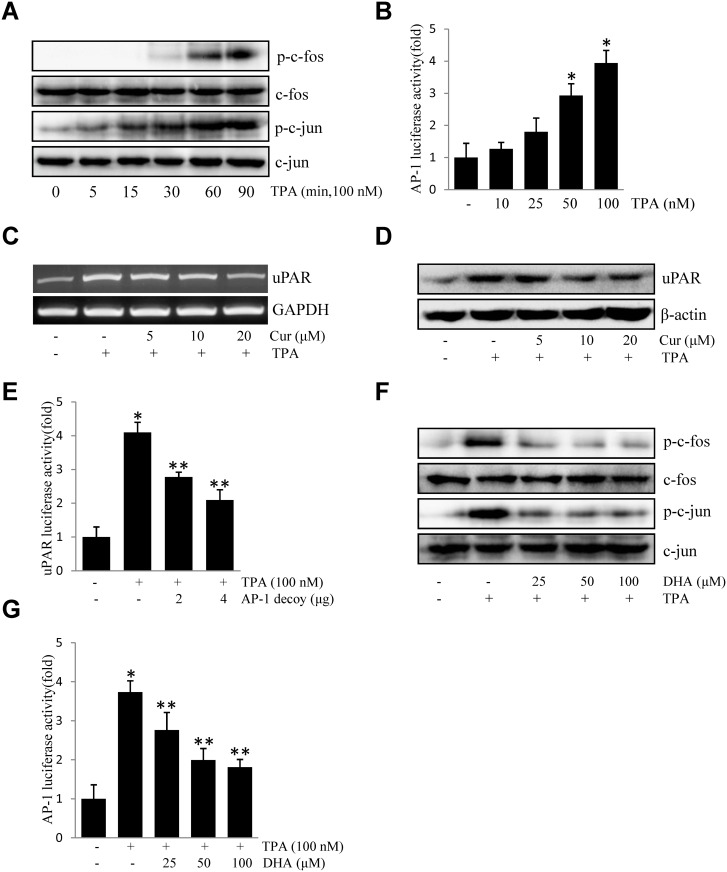
DHA inhibits TPA-induced uPAR by suppressing the DNA-binding activities of AP-1 in ECV304 cells. (A) Cells were treated with TPA for 0–90 min, and the cellular extracts were blotted using specific antibodies. (B) Cells were transiently transfected with the pAP-1 luciferase reporter construct. The transfected cells were incubated with TPA for 4 h and the luciferase activities were determined using a luminometer. (C) Cells were treated with 0–20 μM curcumin (Cur) for 1 h prior to exposure to 100 nM TPA for 4 h. After incubation, the uPAR mRNA levels in the cell lysates were determined by RT-PCR. (D) Cells were treated with 0–20 μM curcumin (Cur) for 1 h prior to exposure to 100 nM TPA for 16 h. After incubation, the uPAR protein levels were determined western blotting. (E) The AP-1 decoy oligonucleotide was co-transfected with pGL3-uPAR into cells. After incubation with 100 nM TPA for 4 h, the luciferase activities were determined using a luminometer. (F) Cells were treated with DHA (25, 50, 100 μM) prior exposure to 100 nM TPA, and the expressions of phos-c-fos, phos-c-jun were analyzed by western blotting. (G) Cells were transiently transfected with the pAP-1 luciferase reporter construct, after being pretreated with DHA (25, 50, 100 μM), and then were incubated with 100 nM TPA for 4 h. After incubation, the cells were lysed and luciferase activity was determined. *P<0.05 versus control; **P<0.05 versus only TPA. The data represent the mean ± SD from triplicate measurements.

Accumulating evidence showed that AP-1 plays a pivotal role in tumorigenesis [24]. To study the role of transcription factors AP-1 in TPA-induced uPAR expression, the effect of TPA on the activation of AP-1 was investigated in ECV304 cells. As shown in Fig 4A, TPA treatment induced the phosphorylation of c-fos and c-jun, both of which are members of the AP-1 family. Consistently, TPA treatment resulted in an increase in AP-1-dependent transcriptional activity in cells transiently transfected with the AP-1 luciferase reporter construct (Fig 4B). Moreover, treatment of cells with 5–20 μM curcumin, an AP-1 inhibitor, suppressed uPAR mRNA (Fig 4C) and protein expression (Fig 4D). Similarly, when ECV304 cells were transiently transfected with an AP-1 decoy, ODN, TPA-induced uPAR promoter activity was decreased by the decoy oligonucleotide in a dose-dependent manner (Fig 4E). Pretreatment with DHA resulted in significant inhibition of TPA-induced activation of c-fos and c-jun (Fig 4F). AP-1 promoter activity was also identified (Fig 4G). These results suggested that DHA inhibits TPA-induced uPAR through the suppression of AP-1 activation.

### DHA inhibits TPA-induced uPAR by suppressing DNA-binding activities of NF-кB p65

**Fig 5:**
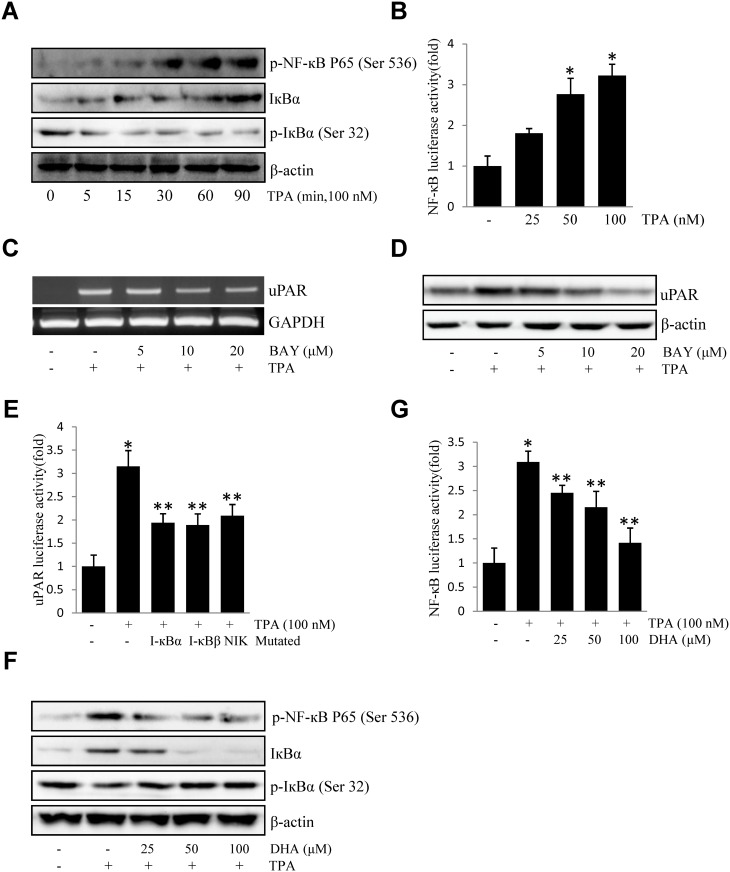
DHA inhibits TPA-induced uPAR by suppressing the DNA-binding activities of NF-кB p65 in ECV304 cells. (A) Cells were treated with TPA for 0–90 min, and the cellular extracts were blotted using specific antibodies. (B) Cells were transiently transfected with the pNF-кB luciferase reporter construct. The transfected cells were incubated with TPA for 4 h and the luciferase activities were determined using a luminometer. (C) Cells were treated with 0–10 μM BAY11-7082 for 1 h prior to exposure to 100 nM TPA for 4 h. After incubation, the uPAR mRNA levels in the cell lysates were determined by RT-PCR. (D) Cells were treated with 0–10 μM BAY11-7082 for 1 h prior to exposure to 100 nM TPA for 16 h. After incubation, the uPAR protein levels were determined by western blotting. (E) The dominant negative mutant of I-κBα, I-κBβ, and NIK were co-transfected with pGL3-uPAR into cells. After incubation with 100 nM TPA for 4 h, the luciferase activities were determined using a luminometer. (F) Cells were treated with DHA (25, 50, 100 μM) prior exposure to 100 nM TPA, and the expressions of phos-p65 (Ser 536), phos-IкB-α (Ser 32), and IкB-α were analyzed by western blotting. (G) Cells were transiently transfected with the pNF-кB luciferase reporter construct, after being pretreated with DHA (25, 50, 100 μM), and then were incubated with 100 nM TPA for 4 h. After incubation, the cells were lysed and luciferase activity was determined. *P<0.05 versus control; **P<0.05 versus only TPA. The data represent the mean ± SD from triplicate measurements.

NF-κB is a pleiotropic, multifunctional transcription factor, involved in cancer proliferation, migration and apoptosis [25, 26]. Activation of NF-κB is usually associated with the induction of IκB phosphorylation; as expected, TPA enhanced the activation of serine 536-phosphorylated NF-κB p65 and serine32-phosphorylated I-κBα and caused the degradation of IκBα in ECV304 cells (Fig 5A). Furthermore, TPA increased transcriptional activity of NF-κB in a concentration-dependent manner (Fig 5B). BAY11-7082 (a NF-κB inhibitor) pretreatment decreased TPA-induced expression of uPAR mRNA (Fig 5C) and protein expression (Fig 5D). Additionally, the expression of dominant negative mutant forms of I-κBα, I-κBβ, or NIK resulted in a decrease in TPA-induced uPAR promoter activity (Fig 5E). Moreover, DHA blocked the activation of serine 536-phosphorylated NF-κB p65 and serine32-phosphorylated I-κBα (Fig 5F) and NF-B promoter activity (Fig 5G). Together, the above data implied that DHA inhibits TPA-induced uPAR through the suppression of NF-κB p65 activation.

### DHA inhibits TPA-induced cell invasiveness

**Fig 6:**
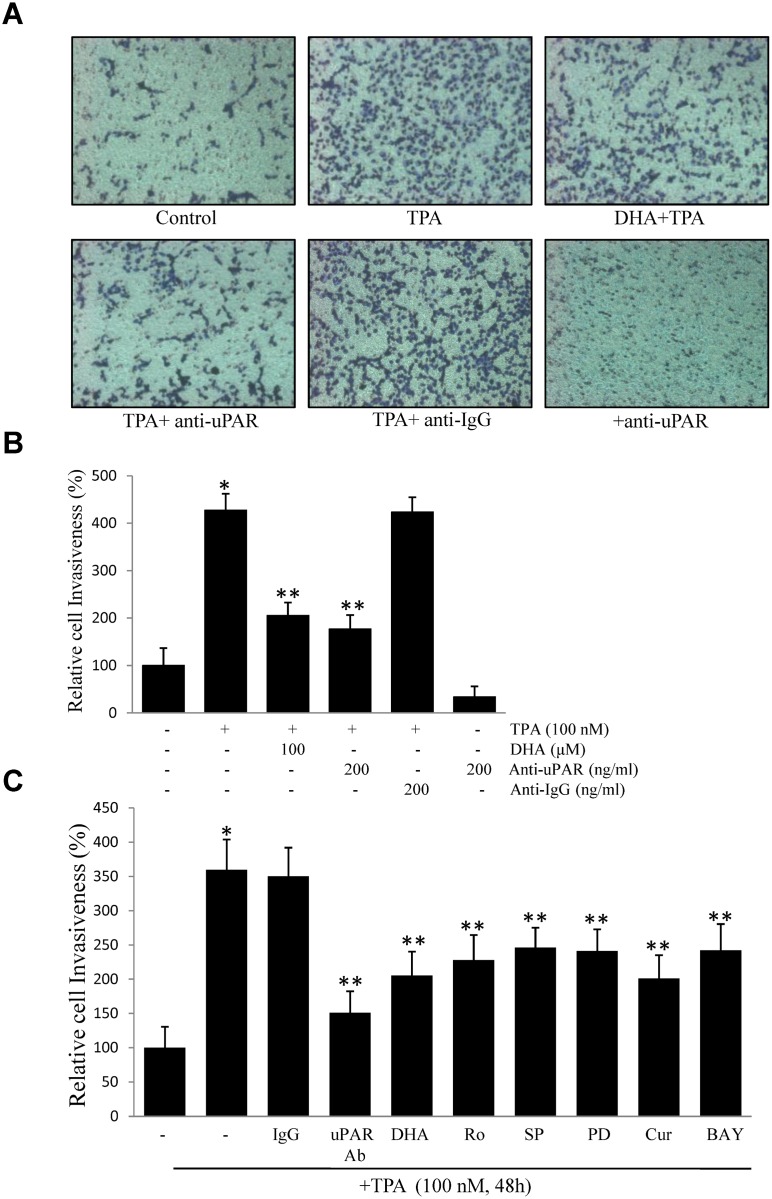
DHA inhibits ECV304 cell invasion by suppressing uPAR. (A) (B) Cells (10^5^) were incubated with 100 nM TPA in the presence or absence of 100 μM DHA or 200 ng/mL uPAR antibody or 200 ng/ml IgG antibody in a BIOCOAT^™^Matrigel apparatus for 48 h. (C) Cells (10^5^) were incubated with 100 nM TPA in the presence of non-specific IgG (200 ng/mL), anti-uPAR antibody (200 ng/mL), 2 μM Ro, 20 μM PD, 20 μM SP, 20 μM Cur, and 20 μM BAY. After incubation, the cells invaded the undersurface of the chambers and were counted using a phase contrast light microscope after staining with a Diff-Quick Stain Kit. *P<0.05 versus control; **P<0.05 versus TPA only. The data represent the mean ± SD from triplicate measurements.

To examine the effect of DHA on TPA-induced cell invasion, we examined cell invasion through a modified Boyden invasion chamber. Incubated of ECV304 cells in TPA resulted in an increased number of invasive cells that passed through the artificial matrigel. However, in the presence of DHA or uPAR antibody, the number of invasive cells decreased, suggesting that DHA may suppress TPA-induced cell invasiveness by inhibiting uPAR expression in ECV304 cells (Fig 6A and 6B). Next, we investigated the effect of a signaling inhibitor on TPA-induced cell invasion. As shown in Fig 6C, uPAR antibody, DHA, Ro, SP, PD, Cur, and BAY inhibited cell invasion induced by TPA, indicating that DHA probably inhibits TPA-induced uPAR via suppression of PKCδ, JNK1/2, and Erk1/2, and reduction of AP-1 and NF-κB activation in ECV304 cells.

## Discussion

Cancer has attracted considerable attention in recent decades, because it is a leading cause of death globally [27]. Much effort has been directed at defining the role of DHA as a cancer chemopreventive agent in humans. This interest has been stimulated by the following observations. i) The ω-3 PUFAs are important constituents of cell membranes that play multiple roles in regulating membrane fluidity, eicosanoid synthesis, cell signaling, and gene expression [28]. Ye *et al* reported that DHA reduces oxidative stress induced calcium influx by altering lipid composition in membrane caveolar rafts [29]. ii) DHA modulates multiple molecular pathways. DHA was reported to activate large-conductance Ca^2+^- dependent K^+^ channels [30]. iii) DHA are natural ligands of several nuclear receptors and transcription factors that regulate gene expression in some tissues [31]. iv) Accumulating evidence indicates that DHA inhibits various genes, including VEGF and COX-2, that are related to inflammation and tumor metastasis [32–34]. v) DHA enhances chemotherapy. In one study, DHA was shown to increase butyrate-mediated apoptosis through promoter methylation [35]. Additionally, DHA is essential for normal brain growth and cognitive function [36]. In this study, we explored the effects of DHA on uPAR expression and cell invasion in ECV304 human endothelial cells. Our results provide novel evidence that DHA effectively inhibits TPA-induced uPAR and cell invasion.

TPA, a protein kinase activator, has been used as a tumor promoter in chemical-induced carcinogenesis in vitro and in vivo. Several studies indicate that up-regulation and activation of PKCs are highly correlated with tumor metastasis [23, 37]. In the present study, using a PKC si-RNA (si-PKC) and PKCδ si-RNA (si-PKCδ), attenuated TPA-induced uPAR, and the ability of DHA to suppress TPA-induced translocation of PKCδ from the cytosol to the plasma membrane may have reduced the metastatic potential. It is noteworthy that PKC degrades with chronic TPA treatment [14], which deserves further rigorous research. MAPKs comprise a highly conserved cascade of serine/threonine kinases connecting cell surface receptors to regulatory targets in response to various stimuli [38]. Pharmacological studies have shown that incubation of ECV304 cells with JNK1/2 inhibitor or Erk1/2 inhibitor attenuated TPA-induced uPAR and that expression of JNK1/2 and Erk1/2 could be diminished by DHA treatment. EGFR is known to play a role in TPA-induced glioblastoma cell proliferation [23, 39]. EGFR was also reported to serve as downstream element in the signaling triggered by uPAR [40]. The Src tyrosine kinase has well established roles in the expression of uPAR and progression of human cancers [41, 42]. In this respect, many additional signaling modulators should be investigated to explore DHA suppression of TPA-induced uPAR and cell invasiveness in ECV304 cells.

Our results agree with earlier reports regarding the role of NF-кB and AP-1 in uPAR expression by macrophage-stimulating protein in gastric cancer AGS cells [43]. AP-1 is composed of members of the c-fos and c-jun families, which have been shown to regulate the expression of a number of genes involved in tumorigenesis. Here, activation of c-fos and c-jun was observed in TPA-treated cells, and they may be key molecules involved in uPAR expression in ECV304 cells. Moreover, DHA’s significant suppression of phosphrylation of c-fos and c-jun accompanied by a reduction in AP-1 transcription factor activity therefore inhibited uPAR expression. The sequestration of NF-κB by IκB in the cytoplasm and IκB phosphorylation leading to the proteasomal degradation of IκBα results in activation and translocation of NF-κB into the nucleus, and it is essential for the expression of several genes [44]. Treatment with DHA attenuated TPA-induced NF-кB DNA binding complex formation, and these results were consistent with a recent study [21]. A prior study suggested that the EGFR signaling activates NF-кB via mTORC2 [45]. The upstream signaling for AP-1 was Erk1/2 and JNK1/2 in cadmium-induced ECV304 cells [46]. It is likely that cross-talk between reactive oxygen species (ROS) and NF-кB might exit and modulate cellular signaling events [47]. Furthermore, the transcription factor SP1 binds to the uPAR promoter [48].

**Fig 7:**
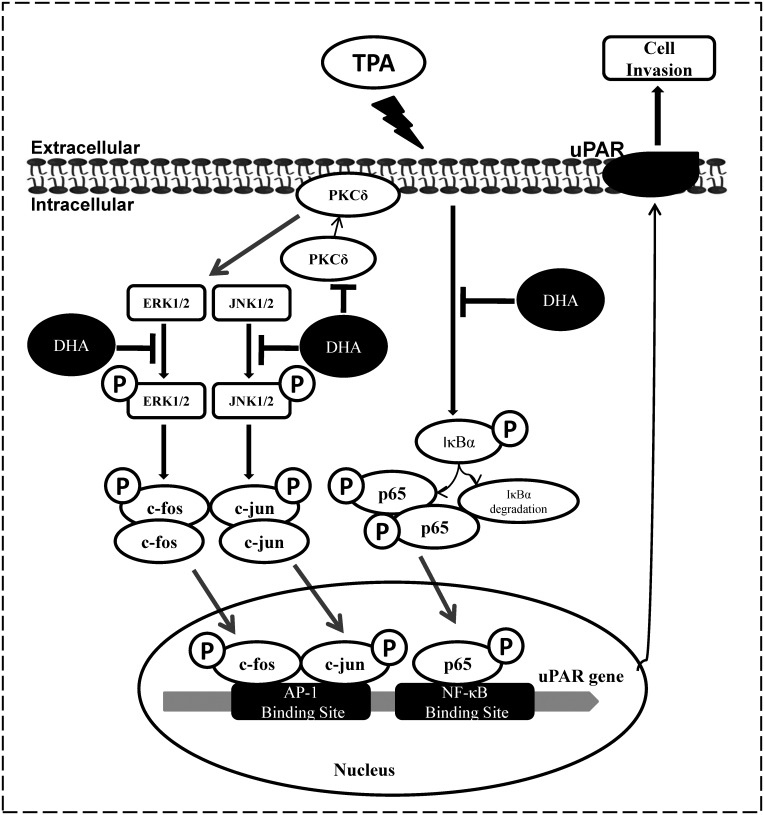
Scheme summarizing the mechanisms of DHA inhibition of TPA-induced uPAR in human endothelial ECV304 cells. DHA inhibits uPAR expression and cell invasion by inhibition of PKCδ, JNK1/2, and Erk1/2, and the reduction of AP-1 and NF-κB activation.

In summary, as shown in Fig 7, our results suggest that DHA inhibits TPA-induced uPAR expression and that it is, at least in part, involved in the inhibition of the PKCδ, JNK1/2 and Erk1/2, signaling pathways and in the reduction of AP-1 and NF-κB transcriptional activation. DHA may represent a novel target molecule or therapeutic approach to repress cancer progression.
